# The Influence of the Partitioning of Sugars, Starch, and Free Proline in Various Organs of *Cyclamen graecum* on the Biology of the Species and Its Resistance to Abiotic Stressors

**DOI:** 10.3390/plants11091254

**Published:** 2022-05-05

**Authors:** John Pouris, Efi Levizou, Maria Karatassiou, Maria-Sonia Meletiou-Christou, Sophia Rhizopoulou

**Affiliations:** 1 Section of Botany, Department of Biology, National and Kapodistrian University of Athens, Panepistimiopolis Athens, 15784 Athens, Greece; jopouris@biol.uoa.gr (J.P.); mmeleti@biol.uoa.gr (M.-S.M.-C.); 2 Department of Agriculture Crop Production and Rural Environment, University of Thessaly, Fytokou Str., 38446 Volos, Greece; elevizou@uth.gr; 3 Laboratory of Rangeland Ecology (PO 286), School of Forestry and Natural Environnent, Aristotle University of Thessaloniki, 54124 Thessaloniki, Greece; karatass@for.auth.gr

**Keywords:** *Cyclamen graecum*, geophyte, Mediterranean, phenology, seasonality

## Abstract

The geophyte *Cyclamen graecum* is native to the eastern Mediterranean. Its beautiful flowers with upswept pink petals appear during early autumn, after the summer drought period and before leaf expansion in late autumn. The floral and leaf development alternates with their cessation in early winter and late spring, respectively. Ecophysiological parameters and processes underlining the life-cycle of *C. graecum* have not previously been published. Seasonal fluctuations of sugars, starch, and free proline have been investigated in tubers, leaves, pedicels, and petals, as well as petal and leaf water status. At the whole plant level, the seasonal co-existence of leaves and flowers is marked by an elevated soluble sugar content, which was gradually reduced as the above-ground plant parts shed. The sugar content of petals and pedicels was lower than that of leaves and tubers. Leaf starch content increased from late autumn to spring and was comparable to that of tubers. The starch content in petals and pedicels was substantially lower than that of tubers and leaves. In tubers, monthly proline accumulation was sustained at relatively constant values. Although the partitioning of proline in various organs did not show a considerable seasonal variation, resulting in an unchanged profile of the trends between tubers, leaves, and flowers, the seasonal differences in proline accumulation were remarkable at the whole plant level. The pronounced petal proline content during the flowering period seems to be associated with the maintenance of floral turgor. Leaf proline content increased with the advance of the growth season. The values of leaf relative water content were sustained fairly constant before the senescence stage, but lower than the typical values of turgid and transpiring leaves. Relationships of the studied parameters with rainfall indicate the responsiveness of *C. graecum* to water availability in its habitat in the Mediterranean ecosystem.

## 1. Introduction

The geophytes exhibit a life-cycle associated with temporal separation of the vegetative phase from the flowering phase and possess perennial tubers, which support their annual growth [1,2,3]. *Cyclamen* L. (Primulaceae) is commercially important and a very popular horticultural genus native to the area around the Mediterranean Basin [4,5,6]. Wild cyclamens are perennial geophytes of woods and rocky areas. *Cyclamen* was mentioned as kyklaminos (κυκλάμινος) in Theophrastus’ writings (4th century BC) [2,7]. The Cyclamen Society [8] recognizes 20 *Cyclamen* species. The cultivated cyclamens are usually hybrids of the spring flowering species *Cyclamen persicum.*

*Cyclamen graecum* Link grows in the wild; it is a perennial, tuberous geophyte, naturally distributed in bushy, stony, and sunlit ground in southern parts of the mainland of Greece and Aegean islands, as well as in coastal areas of Turkey and Cyprus, sometimes being confined into crevices in rocks. Environmental conditions, especially temperature, control annual development and florogenesis in geophytes [9]. *C.* *graecum* is an autumn flowering geophyte. Floral pedicels and leaf petioles arise from the upper part and/or the crown of the over summering, large, perennial tubers that have nodes marked by small buds [10,11]. It has been published that *Cyclamen* species with more than 30 chromosomes, e.g., *C. persicum* (2n = 48) and *C. graecum* (2n = 84), can develop very large tubers [12,13]. Actually, cyclamens do not produce sister tubers, but their tubers enlarge with age [14,15]. In *C. graecum*, thick anchor roots are developed between fibrous roots, from the center of the base of the tuber [8], but their role is not yet fully clarified; it is expected that they penetrate deep into the texture of stony substrate [4,5], which is a habitat of distinct seasonal aridity and moistness. The dark green, heart-shaped leaves of *C. graecum* appear in November. The leaves are slightly angular with variegated green spots on the adaxial surface; parts of the adaxial leaf surface are light green and other parts are dark green, while the abaxial leaf surface is violet-mauve.

Partitioning of total sugars between plant parts is directly linked to developmental flux of carbon molecules, osmolytes, and energy [16]. Moreover, soluble sugars and proline play pivotal roles in plants’ stress responses [16,17,18]. In geophytes, the mobilization of total sugars and starch from below-ground organs generates crucial metabolites to support vegetative and reproductive growth, and synthesize essential compounds. Starch as a storage compound is easily hydrolyzed to soluble sugars that can be transported to growing plant organs. Subsequently, after leaf shedding, total sugars and starch accumulate in below-ground plant parts to sustain metabolism and serve as transient energy storage during the oncoming above-ground vegetative stage [16]. It may be worth noting that sowbread (*panis porcinus*), a common name for the genus *Cyclamen*, is a reference to tubers supposedly being a favorable food for pigs [19,20].

Proline has proven to be a multi-functional tool in plant metabolism during the last two decades. Beyond its well-established roles as an osmoticum and generally a protectant against abiotic stresses [21], its involvement in various developmental processes has also been recognized [18,22]. Its contribution to stress adaptation extends from the protection of photosynthetic apparatus and the involved enzymes to the stabilization of redox balance in the chloroplast, along with reactive oxygen species detoxification. Proline’s involvement in re-adjustment of growth once the stress is relieved relates to its catabolism to support new growth with energy [22]. Under normal non-stress conditions, proline acts as a metabolic signal impacting plant growth and development via regulating metabolite pools and the expression of several genes [18]. Moreover, the widespread phenomenon of proline accumulation in reproductive organs has been attributed to its role as a developmental regulator and flowering signal [18,23]. Interestingly, proline-rich floral nectar has been found in ornamental tobacco flowers and connected with the attraction and reward of visiting pollinators with an energy source to sustain insect flight [24].

The objective of this study was to identify and compare the partitioning of soluble sugars, starch, and proline among above- and below-ground plant parts of Cyclamen graecum, which are exposed to fluctuating environmental conditions, as well as the water status of petals and leaves, which according to the best of our knowledge has not hitherto been published, in order to evaluate ecophysiological traits of tissues exposed to ambient conditions.

## 2. Results

### 2.1. Phenological Stages

**Figure 1:**
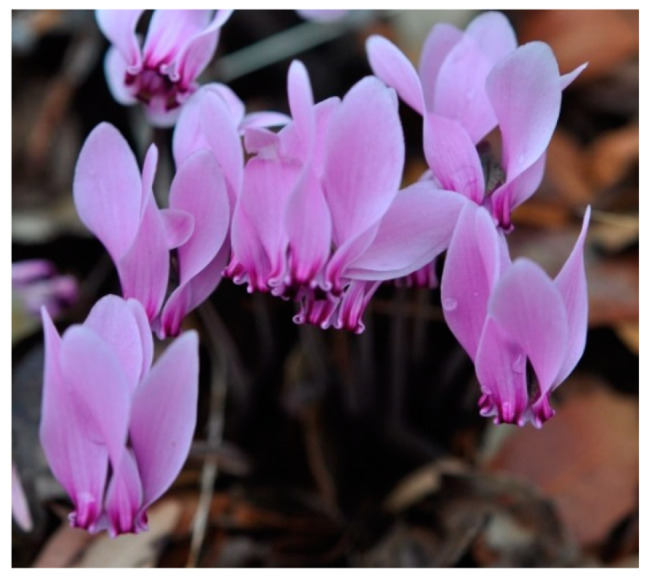
The flowers of *C. graecum* that attract the eye consist of five upswept petals.

**Figure 2:**
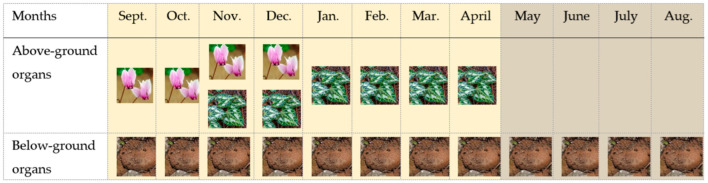
Iconographic presentation of the annual cycle and the phenological stages of *C. graecum* (using illustrations of flowers (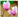), leaves (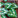) and tubers (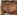)), i.e., the active phase indicated by the ivory area that includes flower initiation and longevity (September–December), and leaf emergence, development, longevity, and senescence (November–April), and the dormant phase indicated by the gray area that includes only the subterranean tubers, because above-ground growth is not visible.

Within the context of seasonality, the life-cycle of *C. graecum* is characterized by two phenological stages in the course of a year: the active phase (from flower emergence in September to leaf senescence in April) and the dormant phase spanning the prolonged drought period (from May to August) in the eastern Mediterranean, when the aboveground plant parts are not visible. Concerning the active phenological stage, the flowers splay open in September (Figure 1 and Figure 2) before leaf emergence in November (Figure 2). The seasonal floral and leaf development of *C. graecum* in early and late autumn (Figure 2) respectively alternates with cessation of flowers and leaves in winter and before summer, respectively. There is a marked variation in the length of the period of flowering and leafing that affects the capacity of this species for resource acquisition.

### 2.2. Sugars

**Figure 3:**
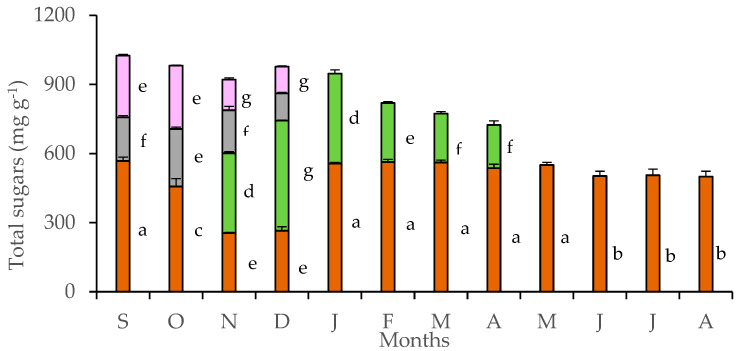
Sugar content in tubers (brown bars), leaves (green bars), pedicels (gray bars), and petals (purple bars) of *Cyclamen graecum*, from September (S) to August (A). Each column denotes means of five replicates ± standard error. Standard Errors smaller than the line thickness of the columns are not shown. Significant differences (*p* < 0.05) of mean values are marked using lowercase letters.

Tubers accumulated large quantities of soluble sugars throughout the year (Figure 3). A gradual reduction of stored sugars in the tubers was detected in October, during the flowering stage, and continued during November and December, when flowers and leaves co-existed. Sugar content of petals and pedicels was lower than that of leaves and tubers, reaching maxima in October. Strong monthly fluctuation of soluble sugars in leaves was detected from November to April, with the maximum values being achieved by mature leaves of December. The considerable sugar content, which was estimated in tubers, declined during the coexistence of floral and leaf stage in November and December. At the whole plant level, the co-existence of leaves and flowers marked the highest sugar content, which was gradually reduced as the above-ground plant parts shed, eventually entering the dormant phase. Monthly values of petal sugar content were positively related with those of tubers (y = 0.523x − 3.882, R² = 0.889, *p* < 0.05) and pedicels (y = 1.227x − 30.461, R² = 0.609, *p* < 0.05), while leaf sugar content was negatively related with that of tubers (y = −0.494x + 337.030, R² = 0.591). In addition, the seasonal rainfall was negatively correlated with the sugar content of petals (y = −0.213x + 87.849, R² = 0.619, *p* < 0.05) and positively correlated with the sugar content of leaves (y = 0.098x + 17.322, R^2^ = 0.709, *p* < 0.05).

### 2.3. Starch

**Figure 4:**
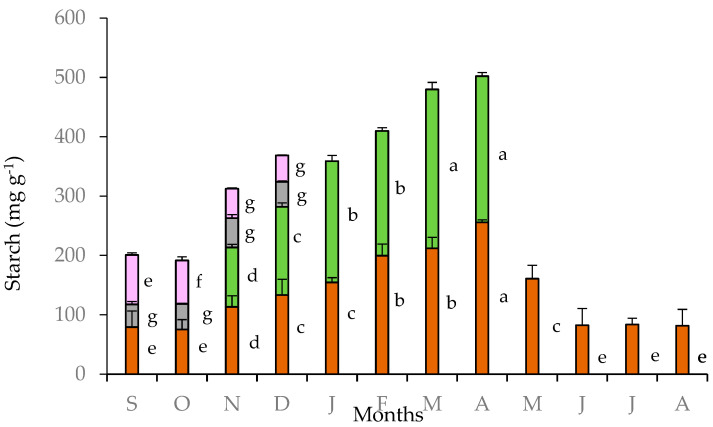
Starch content in tubers (brown bars), leaves (green bars), pedicels (gray bars), and petals (purple bars) of *Cyclamen graecum,* from September (S) to August (A). Each column denotes means of five replicates ± standard error; Standard Errors smaller than the line thickness of the columns are not shown. Significant differences (*p* < 0.05) of mean values are marked using lowercase letters.

The highest starch content in tubers of *C. graecum* was observed in April (256 mg g^−1^) when the climatic conditions were favorable for photosynthesis in the Mediterranean ecosystems, and before the cessation of foliar growth of *C. graecum*. Thereafter, from May to October, starch content declined (Figure 4). The lowest values of starch content in tubers (80 mg g^−1^) were measured from June to October; actually, this period coincides the three-month summer drought (June to August), the cessation of foliar growth, and the early stage of flower formation in September, before the development of new expanding leaves in November (Figure 2). Intermediate values of starch content were detected in tubers from November to January during the development of new leaves. The values of starch content in leaves increased from November to April and were comparable to those of the tubers (Figure 4); in fact, seasonal variation of leaf starch content was positively related with that of tubers (y = 1.027x + 13.422, R² = 0.784, *p* < 0.05). The starch content in petals and pedicels was substantially lower than that of tubers and leaves during the flowering stage (from September to December) (Figure 2). The relatively elevated values of starch content in petals in September and October decreased in November and December (Figure 4), coinciding with elevated starch content in leaves (approximately from 100 to 140 mg g^−1^ d.w.). Negative relationships were detected between petal starch content with the corresponding tuber starch content (y = −0.632x + 125.930, R² = 0.892, *p* < 0.05) and the precipitation in the study site (y = −1.146x + 117.010, R² = 0.839, *p* < 0.05).

### 2.4. Proline

**Figure 5:**
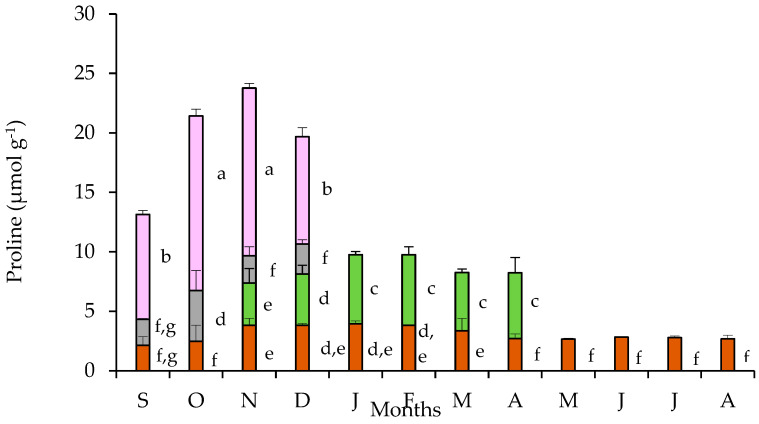
Proline content in tubers (brown bars), leaves (green bars), pedicels (gray bars), and petals (purple bars) of *Cyclamen graecum*, from September (S) to August (A). Each column denotes means of five replicates ± standard error; Standard Errors smaller than the line thickness of the columns are not shown. Significant differences (*p* < 0.05) of mean values are marked using lowercase letters.

The partitioning of free proline in tubers, leaves, pedicels, and petals are presented in Figure 5. The tubers sustained an almost stable free proline content throughout the year, with a slight but statistically significant increase during the cold months (November to March). The pronounced free proline accumulation in petals was evident during the flowering stage showing a substantial enhancement during October and November, the period of full blossom. The pedicels contained a quantity of proline at a comparative level with that of the tubers. The proline content found in leaves during the first two months of their appearance is comparable with that of tubers, but it significantly increased during the next four months of the leaf-stage for this species. Accordingly, the partitioning of proline in the various plant parts did not show a considerable seasonal variation resulting in an unchanged profile of the trends between tubers, leaves, and flowers. Nevertheless, seasonal differences in proline accumulation were obvious at the whole plant level, with the contribution of the proline-rich petals being remarkable.

### 2.5. Water Status of above-Ground Plant Parts

Low values of petal water potential (Ψ) were detected in September (Table 1), after the summer drought period. In October Ψ, osmotic potential (Ψ_s_) and turgor (Ψ_p_) values were increased (less negative) (Table 1), and the same holds true in November. In December, the elevated values of Ψ and Ψ_s_ coincided with the lowest values of Ψ_p_ (Table 1), most probably due to cease of flowering by late December. Regarding Ψ, Ψ_s_, and Ψ_p_ values of petals, significant differences (*p* < 0.05) were found among sampling dates (Table 1).

In petals, significant positive correlations were detected between Ψ and rainfall (y = 0.0123x − 1.2499, R^2^ = 0.954, *p* < 0.05) and Ψ_s_ (y = 0.0122x − 1.3865, R^2^ = 0.811, *p* < 0.05); also, negative relations were detected between soluble sugars and Ψ (y = −0.0029x − 0.1193, R^2^ = 0.730, *p* < 0.05), and Ψ_s_ (y = −0.0034x − 0.6634, R^2^ = 0.843, *p* < 0.05), as well as between starch and Ψ (y= −0.0149x + 0.2407, R^2^ = 0.908, *p* < 0.05) and Ψ_s_ (y = −0.0164x + 0.1905, R^2^ = 0.936, *p* < 0.05), while a positive relationship between Ψ_p_ and proline content was found (y = 0.022x − 0.1168, R^2^ = 0.822, *p* < 0.05), as well as Ψ_p_ and soluble sugars (y = 0.0007x + 0.00146, R^2^ = 0.785, *p* < 0.05).

The relative water content (RWC) of leaves was sustained fairly constant until March and declined in April (Table 2). Additionally, a positive relationship was detected between RWC and rainfall (y = 0.2768x + 63.088, R^2^ = 0.835, *p* < 0.05).

## 3. Discussion

The seasonal accumulation of starch and soluble sugars in the tubers of *C. graecum* confirms to their role in storing photoassimilates and providing a supply of energy to drive new growth [17,25]. The distribution between soluble carbohydrates and starch differed between leaves and tubers. The partitioning of starch to tubers was reasonably similar to that of leaves and a positive linear relationship was detected between tubers and leaves (y = 1.027x + 13.422, R² = 0.784, *p* < 0.05); starch partitioning may be linked to source-sink relationships; the photosynthetically active leaves (the source) provide assimilated carbon (available for transport) to a storage organ (sink), which will utilize it to support metabolic requirements. Furthermore, the elevated values of starch from February to April in both tubers and leaves coincide with the leaf photosynthetic efficiency, and the mild winter temperatures in the Mediterranean area that favor photosynthetic rates. It seems likely that in *C. graecum*, starch may represent a commitment of resources that are acquired by the above-ground tissues simultaneously with the growth of those drawn from stored reserves. This finding is in contrast to data published elsewhere for the summer flowering geophyte *Pancratium maritimum*, where starch was mainly stored in underground bulbs [26].

At the phenological stage of flowering, when the ambient temperatures begin to fall, the concentration of soluble sugars in the tubers rapidly decreased to support the metabolically demanding reproductive growth with carbon source and energy [27,28]; this is also indicated by the positive linear relationship between sugar content between tubers and petals (y = 0.523x − 3.882, R^2^ = 0.881, *p* < 0.05). Floral growth during the early autumn takes precedence over allocation. A further decrease of soluble sugars in tubers at the period of leaf emergence indicates a translocation of stored sugars to sustain leaf development and floral exhibition, when winter lies ahead, via a transition from sink to source. The leaf sugar content during winter and spring denotes an active photosynthetic machinery for this species grown under ambient, environmental conditions, coinciding with the values of leaf RWC before wilting; leaf RWC may also be considered as a measure of the relative cellular volume, affecting interactions among macromolecules. Usually, levels of RWC below 70% imply a water potential at the order of −1.5 MPa or less, and this would cause changes in the metabolism with ceasing of photosynthesis [29], concomitantly with leaf senescence in April. Leaf sugar content may also be associated with the argument that leaves of cyclamen species are a static export pool of sucrose, and the sugar transport is probably linked to a time lag in the export of newly fixed carbon from leaves and low velocity of phloem transport [30]. Concerning the sugar partitioning, some geophytes seem to follow a pattern of relatively higher sugar concentration in the subterranean organs compared to leaves and in some cases, with flowers [31]. In petals, the reduced osmotic potential was significantly related with increased soluble sugar content (y = −0.0034x − 0.1663, R^2^ = 0.834, *p* < 0.05), presumably contributing to turgor maintenance, expansion and water status of these tissues [32,33,34]. Actually, anthesis appears to be due to a pulsed increase in the concentration of soluble sugars [35,36,37,38]. A relationship linked to transfer of sugars between leaves and petals, which might be interesting, was not evaluated, because in *C. graecum*, flower and leaf development are concomitantly exhibited only during a two-month period, i.e., November and December.

Proline content in tubers of *C. graecum* showed a small but statistically significant increase from November to March, compared to the rest of the year. This accumulation may be driven by the low temperatures of the corresponding months and may be considered a stress-related response. An analogous profile was followed by its leaves, resulting in unchanged partitioning of proline between leaves and tubers, when leaves are coming through during the life-cycle of C. graecum; nevertheless, the greatest variation was found between petal and leaf concentrations. It has been published that *C. graecum* is a cold-tolerant species [37,39]. The increased proline biosynthesis and accumulation may partly account for a cold hardiness feature in both tubers and leaves, due to its protectiveness regarding stress and radical scavenging role [40]. Concerning the latter, proline has been related to scavenging of hydroxyl radicals (OH) and possibly other ROS [41], while indirectly modifies the plant’s antioxidant response through increasing the capacity of the involved enzymes, especially ascorbate peroxidase [22]. Additionally, proline accumulation patterns may have implications on nitrogen storage and partitioning, especially under stress conditions [22,27,42]. Proline pool has been reported to expand during transition phase, i.e., from vegetative to reproductive growth [43,44,45,46].

Ecophysiological traits of plant organs that are seasonally either renewed or shed may be a suitable criterion of plant’s adaptation to environmental conditions. *C. graecum* survives the hot summer in a state of dormancy. *C. graecum* blooms in autumn, before leaf emergence. Thereafter, leaves grow and accumulate metabolic reserves throughout the wet and cool season, until the dormancy period, which begins in late spring. In geophytes, long days can initiate the transition to bud dormancy [47,48]. The summer dormancy protects plants from negative effects of water shortage and elevated temperatures on vegetative and reproductive organs, and forces their active development in a more favorable season. *C. graecum* is released from dormancy when the ambient temperatures decrease; hence, shifts from the vegetative to reproductive stage, and floral initiation and differentiation occur in the mature tubers [8,49].

Free proline accumulation in petals was remarkable and significantly increased compared to the other plant organs. Flowers exhibited a 2.5- to 5-fold enhanced proline content in comparison to tubers and 2- to 3-fold compared to leaves. The transportation of proline into the reproductive organs, even under non-stress conditions, has been repeatedly reported [49]. Corroborating our results, a 60-fold higher proline concentration was detected in tomato flowers than in all the vegetative tissues [50]. Enhanced proline content was also documented in petals of *P. maritimum* and was attributed to a requirement for osmotic adjustment, because this geophyte is exposed to dry and saline ambient environmental conditions [26,46]. Multiple explanations of proline accumulation in flowers have been published. For example, the increased proline content has been connected to the high yield of ATP resulting from its oxidation, thus considering proline a molecule well-suited to sustain high energy-demanding processes in reproductive tissues [44]. Low values of petal osmotic potential coincided with enhanced proline accumulation. Additionally, the protective role of proline, which was positively correlated with the turgor of petals of *C. graecum*, has been highlighted during floral developmental processes that include dehydration, as spontaneously occurring during pollen formation or embryogenesis [51,52]. Furthermore, proline may provide a convenient source of energy and nitrogen during immediate post-stress metabolism [46,49,53].

## 4. Material and Methods

### 4.1. Research Site and Plant Phenology

**Figure 6:**
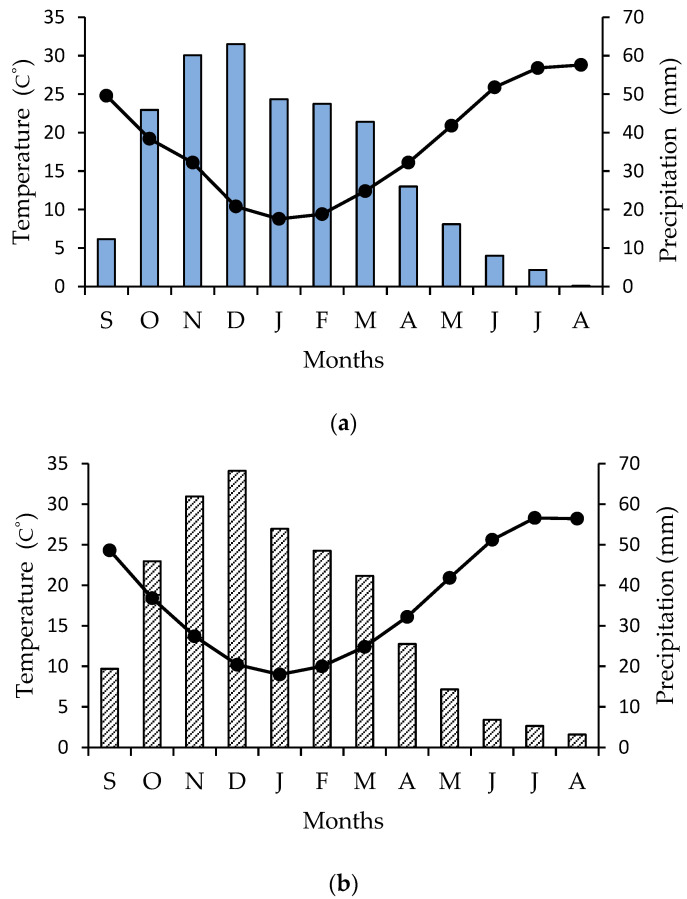
(**a**) Ombrothermic diagram (Precipitation scale = 2 × Temperature scale) for the study site; the order to months is from September (S) of 2018 to August (A) of 2019. Mean monthly precipitation is indicated by blue bars and mean monthly temperature by closed circles and the black line; (**b**) A multiannual ombrothermic diagram (Precipitation scale = 2 × Temperature scale) for the study site from September of 1955 to August of 2010; the order to months is from September (S) to August (A). Mean monthly precipitation is indicated by shaded bars, and mean monthly temperature by closed circles and the black line.

The study was conducted in naturally occurring patches of *Cyclamen graecum* Link distributed in the campus of the National and Kapodistrian University of Athens in Greece (latitude: 37.9664, longitude: 23.756971, altitude 260 m), at the foothills of Hymettus Mountain, where travertine limestone appears along discontinuities of strongly fractured gray dolomite limestone; also, father soil characteristics, texture, and composition have been previously published [54,55]. Concerning perennial geophytes, most bulbs and tubers must reach a critical size before floral induction can occur [56]. In addition, large bulbs and tubers generally produce vigorous above-ground organs and/or many flowers. Therefore, the active and the dormant phenological stage (Figure 2) were monitored, via detailed field observations in distinct niches of *C. graecum* [57], on a monthly basis for two consecutive years (2017 and 2018). The selected at random plants were growing under natural conditions; tubers, leaves, and flowers of *C. graecum* were sampled from a single stand of *C. graecum* surrounded by uniform Mediterranean phryganic vegetation [54], at monthly intervals during the course of a year, i.e., from September of 2018 to August of the next year (2019). The first flowers of *C. graecum* [58] appear in September, while the heart-shaped leaves are coming through in November, arising from the crown of the tubers [59]. Values of mean monthly precipitation and temperature, obtained from a meteorological enclosure, provided by the National Observatory of weather conditions in Greece, are presented in Figure 6a (annual data during the study period) and Figure 6b (multiannual data).

### 4.2. Determination of Total Soluble Sugar and Starch

Soluble sugars were extracted from dry, finely powdered samples (leaves, tubers, pedicels, petals) that were placed in 10 mL 80% ethanol (*v*/*v*), in a shaker, and the extracts were filtered using Whatman # 2 filter paper. Soluble sugar concentration was investigated colorimetrically, according to a modified phenol-sulphuric acid method [60,61], at 490 nm, using a spectrophotometer (Novaspec III^+^ Spectrophotometer, Biochrom, Cambridge, UK). The determination of starch was made in the residue after the extraction of sugars, using the anthrone method [26,62]. D-glucose (Serva, Heidelberg, Germany) aqueous solutions were used for the standard curve. The values are expressed as mg g^−^^1^ d.w.

### 4.3. Determination of Proline

Free proline content was determined colorimetrically on 4 mL samples of the condensed fluid extracted from the plant material [63,64]. The extraction procedure from plant samples (finely powdered dried tubers, leaves, pedicels, and petals) and colorimetric determination were carried out as we have analytically published [26]. Dried, powdered samples were homogenized with aqueous sulphosalicylic acid (20 mL, 3% *v*/*v*), and the homogenate filtered through Whatman # 2 filter paper; 2 mL of the filtrate reacted with acid-ninhydrin solution (2 mL) and glacial acetic acid (2 mL) in test tubes, which were placed in a water bath at 100 °C for 1 h, and the reaction terminated in an ice bath. After cooling, the reaction mixture was extracted with 4 mL toluene and homogenized in a vortex. The chromophore containing the toluene was aspirated from the aqueous phase and the absorbance was read at 520 nm; immediately after, the terminated reaction in glass tubes placed in an ice bath, using toluene as a blank sample and the spectrophotometer mentioned in paragraph 5.2. The proline concentration was estimated using a standard curve of relevant L-proline solutions (Serva, Heidelberg, Germany) and calculated on a dry weight basis.

### 4.4. Determination of Water Status

Petal water potential (Ψ) was measured psychometrically on 6 mm diameter fresh discs (five replicates) from fully expanded petals, which were placed in five C-52 psychrometric chambers (Wescor Inc., Logan, UT, USA) attached to a dew point psychrometer (HR-33T, Wescor Inc.) using the psychrometer switchbox (PS-10, Wescor); the time required for equilibration between the water vapor pressure of leaf sample and that of the psychrometer chamber was 2 h. The osmotic potential (Ψ_s_) was measured using the same leaf discs after freezing and thawing [65,66]. Turgor pressure (Ψ_p_) was calculated as the algebraic difference between Ψ and Ψ_s_. The relative water content (RWC) of fully expanded leaves was determined according to the disc method [29], using the equation RWC (%) = [(FW − DW)/(TW − DW)] × 100, where: FW is the sample fresh weight, TW is the sample turgid weight, and DW is the sample dry weight.

### 4.5. Statistical Analysis

The results are presented as mean ± Standard Error (SE). In order to determine differences in the studied parameters of plant parts of *C. graecum*, a two-way analysis of variance (ANOVA) was performed on the studied parameters at *p* < 0.05 and the Duncan’s multiple range test was applied for comparing the means. All statistical tests were performed using the SPSS statistical v. 23.0 (SPSS Inc., Chicago, IL, USA). Regression analysis was used to determine relationships among results obtained by plant tissues of *C. graecum* and precipitation.

## 5. Conclusions

The life form of *C. graecum* is characterized by two phenological stages in the course of a year, the active phase (from flower emergence in September to leaf senescence in April), and the dormant phase spanning the prolonged drought period, when above-ground plant parts are not exposed to the severity of summer in the eastern Mediterranean. Partitioning patterns of soluble sugars, starch, and free proline in above- and below-ground parts of *C. graecum* contribute to the maintenance of its annual rhythm and phenophases in fluctuating environmental conditions. The remarkable concentration of proline in petals, in comparison to other plant parts during autumn, seems to be associated with the maintenance of their turgor; without turgor, the exposed petals to ambient environmental conditions of the sharply reflexed corolla of *C. graecum* could not be standing so firm and erect. The leaf relative water content was found lower than the typical values of turgid and transpiring leaves; this may indicate that leaves of *C. graecum* subjected to ambient conditions are not susceptible to low temperatures. However, further work will be required to fully test this hypothesis.

## Figures and Tables

**Table 1:** Seasonal water potential (Ψ), osmotic potential (Ψ_s_), and turgor (Ψ_p_) of petals. The values are means of five replicates ± SE.

Months	Ψ (MPa)	Ψ_s_ (MPa)	Ψ_p_ (MPa)
September	−1.08 ± 0.02 ^d^	−1.18 ± 0.05 ^d^	0.10 ± 0.03 ^b,c^
October	−0.74 ± 0.04 ^c^	−0.97 ± 0.02 ^c^	0.23 ± 0.03 ^a^
November	−0.56 ± 0.05 ^b^	−0.72 ± 0.04 ^b^	0.16 ± 0.04 ^b^
December	−0.40 ± 0.02 ^a^	−0.46 ± 0.03 ^a^	0.06 ± 0.02 ^c^

Significant differences (*p* < 0.05) of mean values are marked using lowercase superscript letters that are given separately on each column variable.

**Table 2:** Relative water content (RWC) of leaves. The values are means of five replicates ± SE.

Months	RWC
November	79.34 ± 0.06 ^a^
December	78.83 ± 0.10 ^a^
January	77.33 ± 0.14 ^a^
February	77.15 ± 0.08 ^a^
March	77.23 ± 0.05 ^a^
April	68.38 ± 0.12 ^b^

Significant differences (*p* < 0.05) of mean values are marked using lowercase superscript letters.

## Data Availability

The data are available from the authors upon request.

## References

[1] Dafni A., Cohen D., Noy-Mier I. (1981). Life-cycle variation in geophytes. Ann. Mo. Bot. Gard..

[2] Negbi M. (1989). Theophrastus on geophytes. Bot. J. Linn. Soc..

[3] Akita Y., Ishizaka H., Nakayama M., Shimada A., Kitamura S., Hase Y., Narumi I., Tanaka A. (2010). Comparative analysis of floral pigmentation between wild-type and white-flowered varieties of *Cyclamen graecum*. J. Hortic. Sci. Biotechnol..

[4] Grey-Wilson C. (2015). The Genus Cyclamen.

[5] Mazouz W., Djeddi S. (2013). A biological overview on the genus *Cyclamen*. Eur. J. Sci. Res..

[6] Panitsa M., Trigas P., Kontakos D., Valli A.T., Iatrou G. (2021). Natural and cultural heritage interaction: Aspects of plant diversity in three East Peloponnesian castles (Greece) and conservation evaluation. Plant Biosyst..

[7] Scarborough J. (1978). Theophrastus on herbals and herbal remedies. J. Hist. Biol..

[8] The Cyclamen Society. https://www.cyclamen.org/plants/species/.

[9] Le Nard M., De Hertogh A.A., De Hertogh A.A., Le Nard M. (1993). Bulb growth and development and flowering. The Physiology of Flower Bulbs.

[10] Dole J.M. (2003). Research approaches for determining cold requirements for forcing and flowering of geophytes. Hort. Sci..

[11] Adkins J.A., Miller W.B., Beyl C.A., Trigiano R.N. (2008). Storage Organs. Plant Propagation Concepts and Laboratory Exercises.

[12] Clennett J.C.B. (2002). An analysis and revision of *Cyclamen* L. with emphasis on subgenus *Gyrophoebe* O. Schwarz. Bot. J. Linn. Soc..

[13] Liveri E., Phitos D., Kamari G. (2021). Karyosystematic study of some plant taxa from Greece. Fl. Medit..

[14] Le Nard M., De Hertogh A.A., De Hertogh A.A., Le Nard M. (1993). General chapter on spring flowering bulbs. The Physiology of Flower Bulbs.

[15] Curuk P., Sogut Z., Izgu T., Sevindik B., Tagipur E.M., da Silva J.A.T., Serce S., Solmaz I., Kacar Y.A., Mendi N.Y.Y.Y. (2016). Morphological characterization of *Cyclamen* sp. grown naturally in Turkey: Part II. Acta Sci. Pol. Hortorum Cultus.

[16] Chen L.-Q., Cheung L.S., Feng L., Tanner W., Frommer W.B. (2015). Transport of sugars. Annu. Rev. Biochem..

[17] Ruan Y.-L. (2014). Sucrose metabolism: Gateway to diverse carbon use and sugar signaling. Annu. Rev. Plant Biol..

[18] Szabados L., Savouré A. (2010). Proline: A multifunctional amino acid. Trends Plant Sci..

[19] Harris S. (2008). The Persian Cyclamen. The Temple of Flora Commentary.

[20] Sherwood S., Rix M. (2008). Treasures of Botanical Art.

[21] Ru Q., Wang X., Liu T., Zheng H. (2013). Physiological and comparative proteomic analyses in response to nitrogen application in an Amaryllidaceae plant, *Lycoris aurea*. Acta Physiol. Plant..

[22] Kishor K.P.B., Sreenivasulu N. (2014). Is proline accumulation *per se* correlated with stress tolerance or is proline homeostasis a more critical issue?. Plant Cell Environ..

[23] Mattioli R., Costantino P., Trovato M. (2009). Proline accumulation in plants-Not only stress. Plant Signal. Behav..

[24] Carter C., Shafir S., Yehonatan L., Palmer R.G., Thornburg R. (2006). A novel role for proline in plant floral nectars. Naturwissenschaften.

[25] Kamenetsky R. (2009). Patterns of dormancy and florogenesis in herbaceous perennial plants: Environmental and internal regulation. Crop Sci..

[26] Pouris J., Meletiou-Christou M.S., Chimona C., Rhizopoulou S. (2020). Seasonal functional partitioning of carbohydrates and proline among plant parts of the sand daffodil. Agronomy.

[27] Orthen B. (2001). Sprouting of the fructan-and starch-storing geophyte *Lachenalia minima*: Effects on carbohydrate and water content within the bulbs. Physiol. Plant.

[28] Lundgren M.R., Des Marais D.L. (2020). Life history variation as a model for understanding trade-offs in plant- environment interactions. Curr. Biol..

[29] González L., González-Vilar M. (2011). Determination of relative water content. Handbook of Plant Ecophysiology Techniques.

[30] Rothe K., Porzel A., Neumann S., Grimm E. (1999). Characteristics of the phloem path: Analysis and distribution of carbohydrates in the petiole of *Cyclamen*. J. Exp. Bot..

[31] Li W., Huang D., Wang B., Hou X., Zhang R., Yan M., Liao W. (2022). Changes of starch and sucrose content and related gene expression during the growth and development of Lanzhou lily bulb. PLoS ONE.

[32] Tarpley L., Sassenrath G.F. (2006). Carbohydrate profiles during cotton floral bud (square) development. J. Agron. Crop Sci..

[33] Beauzamy L., Nakayama N., Boudaoud A. (2014). Flowers under pressure: Ins and outs of turgor regulation in development. Ann. Bot..

[34] Rhizopoulou S., Pantazi H. (2015). Constraints on floral water status of successively blossoming Mediterranean plants under natural conditions. Acta Bot. Gall..

[35] van Doorn W.G. (2004). Is petal senescence due to sugar starvation?. Plant Physiol..

[36] van Doorn W.G., Kamdee C. (2014). Flower opening and closure: An update. J. Exp. Bot..

[37] Khodorova N.V., Boitel-Conti M. (2013). The role of temperature in the growth and flowering of geophytes. Plants.

[38] Borghi M., Perez de Souza L., Yoshida T., Fernie A.R. (2019). Flowers and climate change: A metabolic perspective. New Phytol..

[39] Ishizaka H. (1996). Interspecific hybrids of *Cyclamen persicum* and *C. graecum*. Euphytica.

[40] Hajihashemi S., Brestic M., Landi M., Skalicky M. (2020). Resistance of *Fritillaria imperialis* to freezing stress through gene expression, osmotic adjustment and antioxidants. Sci. Rep..

[41] Signorelli S., Arellano J.-B., Melo T.-B., Borsani O., Monza J. (2013). Proline does not quench singlet oxygen: Evidence to reconsider its protective role in plants. Plant Physiol. Biochem..

[42] Ncube B., Finnie J.F., Van Staden J. (2014). Carbon–nitrogen ratio and in vitro assimilate partitioning patterns in *Cyrtanthus guthrieae* L.. Plant Physiol. Biochem..

[43] Tegeder M., Masclaux-Daubresse C. (2018). Source and sink mechanisms of nitrogen transport and use. New Phytol..

[44] Chiang H.H., Dandekar A.M. (1995). Regulation of proline accumulation in *Arabidopsis* during development and in response to dessication. Plant Cell Environ..

[45] Wainwright H., Harwood A.C. (1985). In vitro organogenesis and plant regeneration of *Cyclamen persicum* Mill. using seedling tissue. J. Hortic. Sci..

[46] Carfagna S., Salbitani G., Innangi M., Menale B., De Castro O., Di Martino C., Crawford T.W. (2021). Simultaneous biochemical and physiological responses of the roots and leaves of *Pancratium maritimum* (Amaryllidaceae) to mild salt stress. Plants.

[47] Larcher W. (2003). Physiological Plant Ecology: Ecophysiology and Stress Physiology of Functional Groups.

[48] Proietti S., Scariot V., De Pascale S., Paradiso R. (2022). Flowering mechanisms and environmental stimuli for flower transition: Bases for production scheduling in greenhouse floriculture. Plants.

[49] Ghosh U.K., Islam M.N., Siddiqui M.N., Cao X., Khan M.A.R. (2022). Proline, a multifaceted signaling molecule in plant responses to abiotic stress: Understanding the physiological mechanisms. Plant Biol..

[50] Schwacke R., Grallath S., Breitkreuz K.E., Stransky E., Stransky H., Frommer W.B., Rentsch D. (1999). LeProT1, a transporter for proline, glycine betaine, and gamma-amino butyric acid in tomato pollen. Plant Cell.

[51] Funck D., Winter G., Baumgarten L., Forlani G. (2012). Requirement of proline synthesis during *Arabidopsis* reproductive development. BMC Plant Biol..

[52] Ishizaka H. (2003). Cytogenetic studies in *Cyclamen persicum*, *C. graecum* (Primulaceae) and their hybrids. Plant Syst. Evol..

[53] Stewart C.R. (1973). The effect of wilting on proline metabolism in excised bean leaves in the dark. Plant Physiol..

[54] Margaris N.S. (1976). Structure and dynamics in a phryganic (East Mediterranean) ecosystem. J. Biogeogr..

[55] Kampouroglou E., Economou-Eliopoulos M. (2016). Assessment of the environmental impact by As and heavy metals in lacustrine travertine limestone and soil in Attica, Greece: Mapping of potentially contaminated sites. Catena.

[56] Khalafalla M.M., Menesy F., Magouz M.R., Hamed E.B. (2020). Growth and flowering of endemic wild Libyan geophyte *Cyclamen rohlfsianum* Ascher, with a high ornamental value. Appl. Ecol. Environ. Res..

[57] Yesson C., Culham A. (2006). A phyloclimatic study of *Cyclamen*. BMC Evol. Biol..

[58] Leven S. (2004). Cyclamen graecum. https://www.srgc.org.uk/monthfeature/nov2004/content.html.

[59] Debussche M., Garnier E., Thompson J.D. (2004). Exploring the causes of variation in phenology and morphology in Mediterranean geophytes: A genus-wide study of *Cyclamen*. Bot. J. Linn. Soc..

[60] Dubois M., Gilles K.A., Hamilton J.K., Rebers P.A., Smith F. (1956). Colorimetric method for determination of sugars and related substances. Anal. Chem..

[61] Buysse J., Merckx R. (1993). An improved colorimetric method to quantify sugar content of plant tissue. J. Exp. Bot..

[62] Meletiou-Christou M.S., Rhizopoulou S. (2017). Leaf functional traits of four evergreen species growing in Mediterranean environmental conditions. Acta Physiol. Plant..

[63] Bates L.S., Waldren R.P., Teare I.D. (1973). Rapid determination of free proline for water studies. Plant Soil.

[64] Ain-Lhout F., Zunzunegui M., Barradas M.D., Tirado R., Clavijo A., Novo F.G. (2001). Comparison of proline accumulation in two Mediterranean shrubs subjected to natural and experimental water deficit. Plant Soil.

[65] Richter H. (1978). A diagram for the description of water relations in plant cells and organs. J. Exp. Bot..

[66] Rhizopoulou S., Meletiou-Christou M.S., Diamantoglou S. (1991). Water relations for sun and shade leaves of four Mediterranean evergreen sclerophylls. J. Exp. Bot..

